# Efficacy of Implantable Cardioverter-defibrillators for Secondary Prevention of Sudden Cardiac Death in Patients with End-stage Renal Disease

**DOI:** 10.19102/icrm.2020.110803

**Published:** 2020-08-15

**Authors:** Taylor Payne, Jennifer Waller, Mufaddal Kheda, N. Stanley Nahman, Joyce Maalouf, Aaron Gopal, Haitham Hreibe

**Affiliations:** ^1^Department of Medicine, Medical College of Georgia at Augusta University, Augusta, GA, USA; ^2^Department of Population Health, Medicine Medical College of Georgia at Augusta University, Augusta, GA, USA

**Keywords:** End-stage renal disease, implantable cardioverter-defibrillator, secondary prevention, sudden cardiac arrest, sudden cardiac death

## Abstract

End-stage renal disease (ESRD) constitutes a major burden on the health-care system in the United States, with more than 300,000 patients nationwide being treated with renal replacement therapy. Very few studies to date have evaluated the benefit of implantable cardioverter-defibrillator (ICD) implantation for secondary prevention in patients with ESRD. In this study, we evaluated the efficacy of secondary-prevention ICDs in reducing all-cause mortality in patients on dialysis using the United States Renal Data System (USRDS) database. We queried the USRDS for relevant data between 2004 and 2010. Patients with diagnoses of ventricular fibrillation (VF), ventricular tachycardia (VT), or sudden cardiac arrest (SCA) were included in the study. Patients were excluded from the analysis if they were younger than 18 years; had missing age, sex, or race/ethnicity information; had experienced myocardial infarction; or had an ICD in situ at the time of VF, VT, or SCA diagnosis. The primary endpoint of this study was to determine the efficacy of secondary-prevention ICDs in reducing all-cause mortality in patients on dialysis. A total of 1,442 patients (3.4%) with ESRD had ICD insertion. Patients who received an ICD were predominantly younger, white males with lower Charlson Comorbidity Index and with fewer cardiovascular events. Survival at two years was 53% among those with an ICD relative to 27% among those without an ICD. In this study, we observed a substantial decrease in mortality in patients receiving an ICD for secondary prevention when compared with a cohort of similar patients with a history of VF, VT, or SCA.

## Introduction

End-stage renal disease (ESRD) constitutes a major burden on the health-care system in the United States, with more than 300,000 patients nationwide being treated using renal replacement therapy.^1^ Cardiovascular mortality in patients with ESRD from sudden cardiac arrest (SCA) and arrhythmias accounts for 60% of all of the known documented causes of death in this group.^1^

The efficacy of implantable cardioverter-defibrillators (ICDs) in the secondary prevention [ie, device placed based on demonstration of nonfatal ventricular tachycardia (VT), ventricular fibrillation (VF), or cardiac arrest] of SCA has been demonstrated in the general population^2^; however, no benefit was demonstrated in studies of dialysis patients.

Previous randomized clinical trials have established the efficacy of ICDs in the primary prevention of SCA in the general population but excluded patients with chronic kidney disease.^3–5^ Meanwhile, several retrospective cohort studies and meta-analyses to date have assessed the efficacy of ICDs in the primary prevention of SCA in ESRD patients.^6–21^ In contrast with the findings in the general population, ICD therapy was found to be of little benefit.^6–21^ Moreover, the role of ICDs in the secondary prevention of SCA remains undefined, despite the publication of several small studies.^7,12,13,20,22,23^ On this basis, we hypothesized that the ICD may be of value in the prevention of secondary SCA in a large cohort of dialysis patients. To address this question, we queried the United States Renal Data System (USRDS) for data on the efficacy of ICDs in the prevention of secondary SCA and the associated reduction of all-cause mortality in ESRD patients.

## Methods

### Data source and study population

The USRDS is a de-identified database that includes demographic characteristics, hospital and physician/supplier claims, and vital statistics on all ESRD patients in the United States.^1^ We queried the dataset (hospital claims file) between 2004 and 2010 for all patients who were resuscitated from SCA, defined by the presence of a diagnosis of VF, VT, or SCA [International Classification of Diseases, ninth revision (ICD-9) codes 427.4–427.5]. Variables collected from the patient data file or Centers for Medicare & Medicaid Services form 2728 included age at incident dialysis, sex, race, ethnicity, dialysis modality, and access type. This study was approved by the institutional review board at Augusta University and the need for patient consent was deemed unnecessary.

Patients were excluded from analysis if they were younger than 18 years; had missing age, sex, or race/ethnicity information; had experienced acute myocardial infarction (ICD-9 codes 410–410.92); or had an ICD in place at the time of the SCA diagnosis. Because an instance of VT, VF, or SCA in the setting of an acute myocardial infarction does not meet the criteria for ICD implantation, we determined that patients with these conditions should be excluded from the current study.

ICD implantation following the first SCA diagnosis was determined using ICD-9 diagnosis and Current Procedural Terminology (CPT) procedure codes from the physician/supplier and inpatient detailed claims files. These codes captured device insertion, evaluation, relocation, repair, replacement, repositioning, and/or removal (ICD-9 codes 37.94–37.98, CPT codes 33202–33203, 33215–33218, 33220, 33223–33225, 33230–33231, 33238, 33240–33241, 33243–33244, 33249, 33262–33264, 33270–33273, 93260–93261, 93282–93287, 93289, and 93644). Patients were excluded if they had any of the codes in question reported prior to the SCA diagnosis.

Clinical comorbidities recorded included tobacco use, alcohol use, bacteremia, septicemia, hepatitis B, hepatitis C, cardiovascular events, and pulmonary embolism and were defined using ICD-9 diagnosis codes identified in the hospital claims data. The Charlson Comorbidity Index (CCI), which accounts for the number and severity of various comorbidities, was also determined using hospital claims data.^24^

### Statistical analysis

Descriptive statistics of demographic and clinical risk factors were assessed between patients who received an ICD and those who did not. Continuous variables were expressed as mean ± standard deviation and were compared using two-sample t-tests, while categorical variables were compared using the chi-squared test. The unadjusted chi-squared test and two-sample t-test were used to examine differences between patients with and without ICDs, respectively. Propensity scores for ICD insertion were determined using a logistic regression model and involving independent baseline factors of age, sex, race, ethnicity, dialysis modality, access type, CCI, hepatitis B, hepatitis C, cardiovascular events, tobacco, and alcohol. The propensity score was defined as the probability of ICD insertion and the inverse of the propensity score was used in all additional analyses comparing patients with and without ICDs. A Kaplan–Meier curve with a log-rank test was used to examine preliminary differences in mortality between patients with and without ICDs.

Descriptive statistics on mortality within each risk factor were determined, and chi-squared and two-sample t-tests were used to examine preliminary unadjusted differences between those who died and those who remained alive. To estimate the adjusted hazard ratio (aHR) for mortality associated with ICD insertion, controlling for other demographic or clinical risk factors, a Cox proportional hazards (CPH) model-building strategy was adopted with inverse weighted propensity scores. The final model contained the ICD insertion status and all risk factors that were statistically significant or required in the model to improve the model’s fit. The aHR and corresponding 95% confidence interval (CI) were estimated for each variable in the final model and each aHR was interpreted as the HR for that specific variable while adjusting for all other variables in the final model.

All statistical analyses were performed using SAS version 9.4 (SAS Institute, Cary, NC, USA) and statistical significance was assessed using an alpha level of 0.05.

## Results

### Patient characteristics

A total of 42,519 patients met the screening criteria for SCA with a follow-up period of up to eight years. From this group, 1,442 patients (3.4%) underwent ICD insertion following a SCA diagnosis. The general characteristics of the patients with and without ICDs are shown in **Table 1**. Patients who received an ICD were more often younger at the initiation of dialysis, white, male, showed lower CCI scores, and had fewer cardiovascular events. They were also more likely to have the diagnosis of bacteremia or septicemia in comparison with those without an ICD. There were no differences between the two groups with respect to tobacco or alcohol use, hepatitis B, hepatitis C, or pulmonary embolism. Further, the ICD patients experienced less patient mortality (72.4% versus 79.1%; p < 0.0001) and longer survival times (1.9 ± 1.6 years versus 1.1 ± 1.4 years, respectively; p < 0.001) than those without ICDs. Meanwhile, those with an ICD showed higher rates of death due to a cardiac cause but lower rates of death due to a vascular cause in comparison with those without an ICD.

### Mortality following SCA

**Table 2** presents the demographic and clinical risk factors for mortality in patients with a diagnosis of SCA. Placement of an ICD conferred a significant survival advantage, with 27.6% of ICD patients remaining alive at the end of the follow-up period in comparison with 20.9% of patients without ICDs (p < 0.001). Mortality in patients who survived SCA was associated with female sex, non-Black race, non-Hispanic ethnicity, initiating dialysis with hemodialysis, and initiating dialysis with a catheter or unknown access. In addition, clinical risk factors associated with death included a higher CCI score, septicemia, and pulmonary embolism. Death was less common among patients with tobacco and/or alcohol use, bacteremia, hepatitis B, hepatitis C, cardiovascular events, and potential anticoagulation.

**Figure 1:**
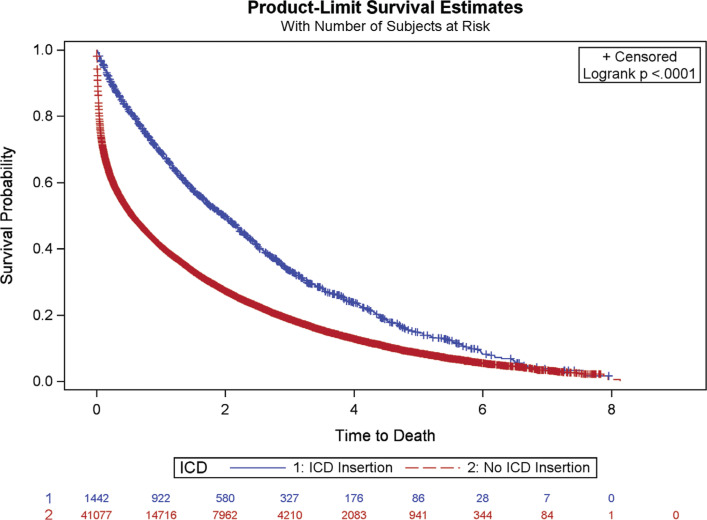
Product-limit survival estimates, with the number of subjects at risk.

**Figure 1** shows the unadjusted Kaplan–Meier survival curve and highlights that that those with an ICD achieved significantly better survival rates than those without an ICD. The survival rates at two years were 53% among those with an ICD and 27% among those without an ICD. The median survival time was 2.19 years (95% CI: 1.96–2.44) among those with an ICD and 0.58 years (95% CI: 0.56–0.60) among those without an ICD.

**Figure 2:**
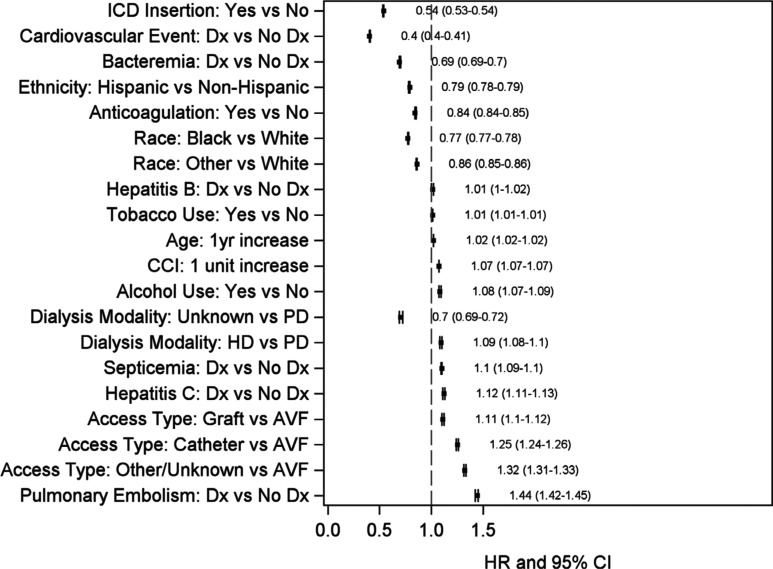
The aHR of ICD insertion and other risk factors for mortality.

In the final adjusted CPH model **(Figure 2)**, ICD insertion was protective for death with an aHR of 0.54. Other protective variables in the entire SCA population included Black versus White race, other versus White race, Hispanic versus non-Hispanic ethnicity, bacteremia, cardiovascular events, and potential anticoagulation. Significant risk factors for death in this group included age at first dialysis, hemodialysis versus peritoneal dialysis, catheter versus arteriovenous fistula (AVF), arteriovenous graft versus AVF, and other including dialysis catheter versus AVF. Other clinical risk factors for death included CCI score, tobacco and alcohol use, septicemia, hepatitis B, hepatitis C, and pulmonary embolism.

### Mortality following ICD placement

**Table 3** indicates the demographic and clinical risk factors for mortality in the 1,442 patients with ICDs. During the study period, 72.4% of the patients with ICDs died. Demographic factors associated with mortality included older age at the initiation of dialysis, White race, non-Hispanic ethnicity, and initiating dialysis with hemodialysis or vascular access with a catheter. Clinical risk factors for death included greater CCI score, tobacco use, and septicemia. There was no significant association with alcohol use, bacteremia, hepatitis B, hepatitis C, cardiovascular events, pulmonary embolism, ICD removal, or potential anticoagulation observed.

**Figure 3:**
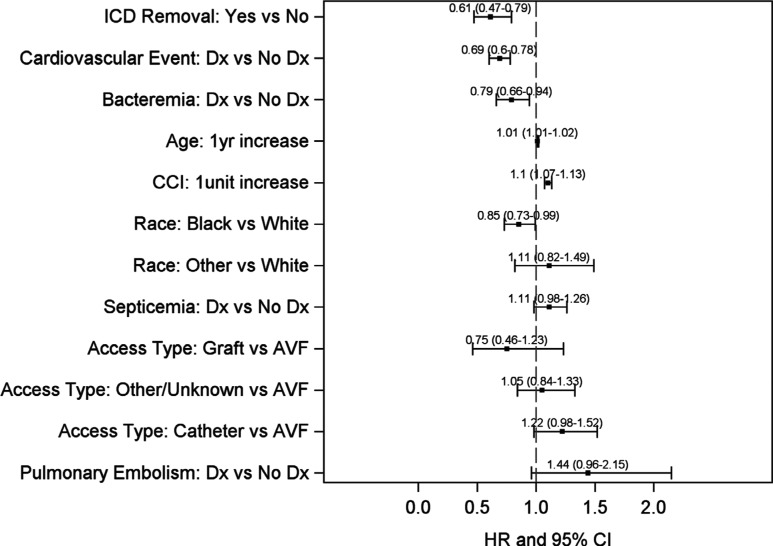
aHR of risk factors for mortality among those with ICD insertion.

**Figure 3** presents the final CPH model for the risk of mortality in patients with ICDs. An increased aHR for death was present for age at first dialysis and CCI score. There was also an decreased aHR for death in Black versus White race, bacteremia, cardiovascular events, and following ICD removal.

## Discussion

In this study, we found a significant decrease in all-cause mortality in patients receiving an ICD implant for secondary prevention of fatal dysrhythmias when compared with a cohort of similar patients without ICDs. In ESRD patients with a history of SCA, ICD insertion is effective for the secondary prevention of fatal dysrhythmias, significantly reducing the aHR for death to 0.54. As a result, the median survival time with an ICD was over threefold longer (2.19 years versus 0.58 years) than in patients without a device.

The present study compared clinical differences between ESRD patients with and without ICDs and found that device insertion is associated with changes in several comorbidities. In this regard, this work highlighted that ICD recipients had lower CCI scores, fewer cardiovascular events, and higher rates of bacteremia than those without ICDs. We speculate that the higher CCI scores in those without ICDs may reflect undesirable comorbidities that precluded surgical intervention and thus excluded patients from undergoing device insertion. The presence of other comorbidities not included in the CCI score may have also impacted the ICD implantation rate. The registry-based aspect of this study is such that these factors cannot be accounted for. Patients with ICDs also had fewer coded cardiovascular events, perhaps due to the greater control of dysrhythmias conferred by the device.

In addition, while ICD recipients exhibited a greater incidence of bacteremia (likely the result of the increased risk for infection conferred by intravascular leads and the presence of the device in the subcutaneous tissues), they did not display a similar rise in septicemia. This might be explained by the more aggressive and earlier protocol for the treatment of infection in this group. The ICD removal rate of 6.4% in our patients was higher than other reported extraction rates in randomized controlled ICD trials in the general population (1.5%–4%).^5,25^ We speculate that the increased incidence of bacteremia underlies this difference. Finally, other studies have shown that ICD recipients with ESRD are more likely to experience infectious complications, adverse bleeding events, lead displacement, and device-related thrombosis relative to otherwise similar ICD recipients without ESRD.^27,28^ Similar to in our work, some studies reported a significant mortality benefit despite these complications.^27,28^

In the present research, patients who received an ICD showed improved two-year survival rates following device placement as compared with those without an ICD. Although these data are comparable with those of other studies, the present work conferred an even greater mortality benefit by comparison.^7,12,29^ We speculate this was due to the higher incidence of arrhythmic events in secondary prevention patients but we cannot exclude that the patients were generally healthier, as suggested by the lower CCI.

The current study suggests an underutilization of ICD in ESRD patients. In this regard, only 3.4% of our patients received an ICD following SCA. This represents a very low implantation rate in a group of patients at high risk of SCA. Moreover, the ICD implantation rate in the general population among survivors of SCA from the National Health Discharge Summary database was 30.7% prior to discharge.^29^ Previous studies of ICD implantation in the ESRD population for secondary prevention also suggested significant underutilization of the procedure, with rates ranging from 8.24 to 38.4%.^28,30^

The possible explanations for the low implantation rate observed in the present study may include the retrospective nature of querying an administrative dataset and/or the presence of clinical contraindications to ICD implantation. In this regard, the utilization of an administrative database may be subject to coding errors for either device placement and/or the accurate classification of an SCA. More likely, clinical contraindications to device placement may be present and could include tachy-dysrhythmias from reversible electrolyte disorders (eg, hyperkalemia), drugs, or unexplained syncopal events erroneously classified as an SCA; no expectation of survival for at least one year; end-stage heart failure; and/or active infection or other medical issues precluding surgical placement of a device. Many of the above clinical issues are common in the ESRD population and we speculate that these problems temper the enthusiasm for ICD placement in some cases. In addition, the median survival in non-ICD recipients was 0.58 years (95% CI: 0.56–0.60) as compared with 2.19 years (95% CI: 1.96–2.44) among ICD recipients. Whether this difference in mortality is fully attributed to ICD implantation or whether anticipated lower survival rates among non-ICD recipients influenced the decision to implant an ICD is difficult to fully evaluate due to the registry-based nature of the study.

The present study demonstrated several significant clinical risk factors for death in ESRD patients following SCA and includes a higher CCI score and systemic infection, with the latter coded as septicemia. Similar associations were found among patients receiving an ICD. In the final models, both groups shared a higher CCI score as a factor significantly increasing the hazard ratio for death. We speculate that the increased burden of comorbidities captured by the CCI is amplified in patients following SCA.

In the final model for survival, the SCA group also showed an increased risk for death from septicemia. In contrast, ICD recipients with bacteremia presented a decreased risk of death. We speculate that ICD patients with bacteremia displayed improved outcomes due to a more aggressive treatment regimen, given the adverse effects of bacteremia in the presence of an ICD. This contention is indirectly supported by the decreased aHR for death following device removal.

The present study has several limitations common to queries of large administrative datasets as we have described.^31^ In brief, all diagnoses and procedures were inferred from billing codes or extracted from forms and were not identified using actual medical documentation. However, the size of the dataset may, in part, offset these limitations. Furthermore, the query was limited to inpatient encounters in which SCA and device presence were coded. Thus, this work cannot account for inpatients in whom query diagnoses were missed or miscoded. In addition, inaccurate diagnoses are impossible to avoid in a retrospective database study of diagnoses that may depend on clinical findings, fostering the potential for bias in the results. Finally, residual confounding in this observational study, due to unmeasured factors that are different between ICD recipients and the SCA control cohorts, is also possible. Although an indication of ICD implantation in secondary prevention is independent of the patient’s left ventricular ejection fraction, the lack of left ventricular ejection fraction data remains a major limitation of this study given the major prognostic impact of left ventricular function as compared with other comorbidities.

In summary and despite the discussed limitations, we have shown, using a large administrative dataset, that ICD placement is likely effective in the secondary prevention of SCA in ESRD patients. Decisions to place an ICD are complicated by medical comorbidities and the increased risk of infection in this cohort but, if successful, ICD placement may be expected to increase the life expectancy by more than threefold as compared with in patients without a device. These data also emphasize the importance of effective collaboration between the nephrologist and the cardiologist when caring for these complex patients.

## Figures and Tables

**Table 1:** Descriptive Statistics by ICD Insertion and Chi-squared or Student’s t-test Results

Variable	Characteristic	ICD (n = 1,442; 3.4%)	No ICD (n = 41,077; 96.6%)	p-value
Demographics				
Age at first dialysis (years), mean ± SD		62.8 ± 12.5	66.4 ± 13.2	< 0.0001
Sex, n (%)	Female	563 (39.0)	21,929 (53.4)	< 0.0001
	Male	879 (61.0)	19,148 (46.6)	
Race, n (%)	Black	392 (27.2)	13,484 (32.8)	< 0.0001
	Other	67 (4.7)	1,555 (3.8)	
	White	983 (68.2)	26,038 (63.4)	
Ethnicity, n (%)	Hispanic	158 (11.0)	4,771 (11.6)	0.4431
	Non-Hispanic	1,284 (89.0)	36,306 (88.4)	
Dialysis modality, n (%)	HD	1,335 (92.6)	38,266 (93.2)	0.5692
	Unknown	16 (1.1)	484 (1.2)	
	PD	91 (6.3)	2,327 (5.7)	
Access type, n (%)	Catheter	797 (55.3)	24,340 (59.3)	0.0035
	Graft	37 (2.6)	1,246 (3.0)	
	Other/unknown	455 (31.6)	11,916 (29.0)	
	AVF	153 (10.6)	3,575 (8.7)	
Clinical diagnosis				
CCI, mean ± SD		5.3 ± 2.4	5.9 ± 2.5	< 0.0001
Tobacco, n (%)	Yes	220 (15.3)	5,935 (14.5)	0.3913
	No	1,222 (84.7)	35,142 (85.6)	
Alcohol, n (%)	Yes	32 (2.2)	769 (1.9)	0.3407
	No	1,410 (97.8)	40,308 (98.1)	
Bacteremia, n (%)	Dx	216 (15.0)	3,771 (9.2)	< 0.0001
	No Dx	1,226 (85.0)	37,306 (90.8)	
Septicemia, n (%)	Dx	610 (42.3)	16,360 (39.8)	0.0593
	No Dx	832 (57.7)	24,717 (60.2)	
Hepatitis B, n (%)	Dx	9 (0.6)	302 (0.7)	0.6266
	No Dx	1,433 (99.4)	40,775 (99.3)	
Hepatitis C, n (%)	Dx	55 (3.8)	1,217 (3.0)	0.0621
	No Dx	1,387 (96.2)	39,860 (97.0)	
Cardiovascular event, n (%)	Dx	559 (38.8)	36,607 (89.1)	< 0.0001
	No Dx	883 (61.2)	4,470 (10.9)	
Pulmonary embolism, n (%)	Dx	33 (2.3)	932 (2.3)	0.9609
	No Dx	1,409 (97.7)	40,145 (97.7)	
ICD removal among those with an ICD, n (%)	Yes	93 (6.4)		
	No	1,349 (93.6)		
Potential anticoagulation, n (%)	Yes	158 (11.0)	3,008 (7.3)	< 0.0001
	No	1,284 (89.0)	38,069 (92.7)	
Mortality, n (%)	Died	1,044 (72.4)	32,508 (79.1)	< 0.0001
	Alive	398 (27.6)	8,569 (20.9)	
Time to death/follow-up (years), mean ± SD		1.9 ± 1.6	1.1 ± 1.4	< 0.0001
Primary cause of death (among those who died)	Cardiac	454 (43.5)	10,551 (32.5)	< 0.0001
	Endocrine	0 (0.0)	5 (0.0)	
	Gastrointestinal	4 (0.4)	218 (0.7)	
	Infection	78 (7.5)	3,355 (10.3)	
	Liver disease	8 (0.8)	123 (0.4)	
	Metabolic	5 (0.5)	188 (0.6)	
	Other	441 (42.2)	13,989 (43.0)	
	Vascular	54 (5.2)	4,079 (12.6)	

Dx: diagnosis; HD: hemodialysis; ICD: implantable cardioverter-defibrillator; PD: peritoneal dialysis; SD: standard deviation.

**Table 2:** Descriptive Statistics for Mortality within the Patient Population and Each Risk Factor and Chi-squared or Student’s t-test Results

Variable	Characteristic	Died (n = 33,552; 78.9%)	Alive (n = 8,967; 21.1%)	p-value
Main independent risk factor				
ICD insertion	Yes	1,044 (72.4)	398 (27.6)	< 0.0001
	No	32,508 (79.1)	8,569 (20.9)	
Demographic risk factors				
Age at first dialysis (years), mean ± SD		67.9 ± 12.7	60.3 ± 13.2	< 0.0001
Sex, n (%)	Female	17,793 (79.1)	4,699 (20.9)	< 0.0001
	Male	15,759 (78.7)	4,268 (21.3)	
Race, n (%)	Black	10,161 (73.2)	3,715 (26.8)	< 0.0001
	Other	1,248 (76.9)	374 (23.1)	
	White	22,143 (82.0)	4,878 (18.0)	
Ethnicity, n (%)	Hispanic	3,623 (73.5)	1,306 (26.5)	< 0.0001
	Non-Hispanic	29,929 (79.6)	7,661 (20.4)	
Dialysis modality, n (%)	HD	31,486 (79.5)	8,115 (20.5)	< 0.0001
	Unknown	270 (54.0)	230 (46.0)	
	PD	1,796 (74.3)	622 (25.7)	
Access type, n (%)	Catheter	19,313 (76.8)	5,824 (23.2)	< 0.0001
	Graft	951 (74.1)	332 (25.9)	
	Other/unknown	10,673 (86.3)	1,698 (13.7)	
	AVF	2,615 (70.1)	1,113 (29.9)	
Clinical risk factor				
CCI, mean ± SD		5.9 ± 2.5	5.8 ± 2.2	< 0.0001
Tobacco, n (%)	Yes	4,471 (72.6)	1,684 (27.4)	< 0.0001
	No	29,081 (80.0)	7,283 (20.0)	
Alcohol, n (%)	Yes	593 (74.0)	208 (26.0)	< 0.0001
	No	32,959 (79.0)	8,759 (21.0)	
Bacteremia, n (%)	Dx	3,091 (77.5)	896 (22.5)	< 0.0001
	No Dx	30,461 (79.1)	8,071 (21)	
Septicemia, n (%)	Dx	14,656 (86.4)	2,314 (13.6)	< 0.0001
	No Dx	18,896 (74.0)	6,653 (26.0)	
Hepatitis B, n (%)	Dx	231 (74.3)	80 (25.7)	0.0444
	No Dx	33,321 (78.9)	8,887 (21.1)	
Hepatitis C, n (%)	Dx	940 (73.9)	332 (26.1)	< 0.0001
	No Dx	32,612 (79.1)	8,635 (20.9)	
Cardiovascular event, n (%)	Dx	28,872 (77.7)	8,294 (22.3)	< 0.0001
	No Dx	4,680 (87.4)	673 (12.6)	
Pulmonary embolism, n (%)	Dx	789 (81.8)	176 (18.2)	< 0.0001
	No Dx	32,763 (78.8)	8,791 (21.2)	
ICD removal among those with an ICD, n (%)	Yes	68 (73.1)	25 (26.9)	0.5639
	No	976 (72.4)	373 (27.7)	
Potential anticoagulation, n (%)	Yes	2,433 (76.9)	733 (23.2)	< 0.0001
	No	31,119 (79.1)	8,234 (20.9)	
Time to death/follow-up (years), mean ± SD		0.8 ± 1.2	2.1 ± 1.6	< 0.0001

CCI: Charlson Comorbidity Index; Dx: diagnosis; HD: hemodialysis; ICD: implantable cardioverter-defibrillator; PD: peritoneal dialysis; SD: standard deviation.

**Table 3:** Descriptive Statistics for Mortality Within Each Risk Factor Category Among Those with ICD Insertion (n = 1,442) and Chi-squared or Student’s t-test Results

Variable	Characteristic	Died (n = 1,044; 72.4%)	Alive (n = 467; 27.6%)	p-Value
Demographic risk factor				
Age at first dialysis (years), mean ± SD		64.6 ± 11.7	58.2 ± 13.4	< 0.0001
Sex, n (%)	Female	400 (71.0)	163 (29.0)	0.3582
	Male	644 (73.3)	235 (26.7)	
Race, n (%)	Black	249 (63.5)	143 (36.5)	< 0.0001
	Other	48 (71.6)	19 (28.4)	
	White	747 (76.0)	236 (24.0)	
Ethnicity, n (%)	Hispanic	106 (67.1)	52 (32.9)	0.1135
	Non-Hispanic	938 (73.1)	346 (27)	
Dialysis modality, n (%)	HD	978 (73.3)	357 (26.7)	0.0314
	Missing/unknown	9 (56.3)	7 (43.8)	
	PD	57 (62.6)	34 (37.4)	
Access type, n (%)	Catheter	557 (69.9)	240 (30.1)	< 0.0001
	Graft	19 (51.4)	18 (48.7)	
	Other/unknown	372 (81.8)	83 (18.2)	
	AVF	96 (62.8)	57 (37.3)	
Clinical risk factor				
CCI, mean ± SD		5.4 ± 2.4	5.2 ± 2.3	0.2889
Tobacco, n (%)	Yes	147 (66.8)	73 (33.2)	0.0443
	No	897 (73.4)	325 (26.6)	
Alcohol, n (%)	Yes	23 (71.9)	9 (28.1)	0.9465
	No	1,021 (72.4)	389 (27.6)	
Bacteremia, n (%)	Dx	162 (75.0)	54 (25.0)	0.3538
	No Dx	882 (71.9)	344 (28.1)	
Septicemia, n (%)	Dx	495 (81.2)	115 (18.9)	< 0.0001
	No Dx	549 (66.0)	283 (34.0)	
Hepatitis B, n (%)	Dx	4 (44.4)	5 (55.6)	0.1256
	No Dx	1,040 (72.6)	393 (27.4)	
Hepatitis C, n (%)	Dx	36 (65.5)	19 (34.6)	0.2401
	No Dx	1,008 (72.7)	379 (27.3)	
Cardiovascular event, n (%)	Dx	414 (74.1)	145 (25.9)	0.2615
	No Dx	630 (71.4)	253 (28.7)	
Pulmonary embolism, n (%)	Dx	25 (75.8)	8 (24.2)	0.6624
	No Dx	1,019 (72.3)	390 (27.7)	
ICD removal, n (%)	Yes	68 (73.1)	25 (26.9)	0.8726
	No	976 (72.4)	373 (27.7)	
Potential anticoagulation, n (%)	Yes	110 (69.6)	48 (30.4)	0.4076
	No	934 (72.7)	350 (27.3)	
Time to death/follow-up (years), mean ± SD		1.7 ± 1.5	2.5 ± 1.7	< 0.0001
Primary cause of death (among those who died)	Cardiac	454 (43.5)		
	Endocrine	4 (0.4)		
	Gastrointestinal	78 (7.5)		
	Infection	8 (0.8)		
	Liver disease	5 (0.5)		
	Metabolic	441 (42.2)		
	Other	54 (5.2)		
	Vascular	454 (43.5)		

CCI: Charlson Comorbidity Index; Dx: diagnosis; HD: hemodialysis; ICD: implantable cardioverter-defibrillator; PD: peritoneal dialysis; SD: standard deviation.

## References

[1] United States Renal Data System. USRDS Annual Data Report 2017: Epidemiology of Kidney Disease in the United States. Volume 2: End-Stage Renal Disease in the United States..

[2] Antiarrhythmics Versus Implantable Defibrillators (AVID) Investigators. (1997). A comparison of antiarrhythmic-drug therapy with implantable defibrillators in patients resuscitated from near-fatal ventricular arrhythmias.. N Engl J Med..

[3] O’Shaughnessy M, Lappin D, Reddan D (2012). Sudden cardiac death in dialysis: do current guidelines for implantable cardioverter defibrillator therapy apply to patients with end-stage kidney disease?. Semin Dialysis..

[4] Moss A, Hall W, Cannom D (1996). Improved survival with an implanted defibrillator in patients with coronary disease at high risk for ventricular arrhythmia.. N Engl J Med..

[5] Moss A, Zareba W, Hall W (2002). Prophylactic implantation of a defibrillator in patients with myocardial infarction and reduced ejection fraction.. N Engl J Med..

[6] Chen T, Wo H, Chang P, Wang C, Wen M, Chou C (2014). A meta-analysis of mortality in end-stage renal disease patients receiving implantable cardioverter defibrillators (ICDs).. PLoS One..

[7] Makki N, Swaminathan P, Hanmer J, Olshansky B (2013). Do implantable cardioverter defibrillators improve survival in patients with chronic kidney disease at high risk of sudden cardiac death? A meta-analysis of observational studies.. Europace..

[8] Hreybe H, Razak E, Saba S (2007). Effect of end-stage renal failure and hemodialysis on mortality rates in implantable cardioverter-defibrillator recipients.. Pace..

[9] Khan F, Adelstein E, Saba S (2010). Implantable cardioverter defibrillators confer survival benefit in patients with renal insufficiency but not in dialysis-dependent patients.. J Interv Card Electrophysiol..

[10] Wase A, Basit A, Nazir R (2004). Impact of chronic kidney disease upon survival among implantable cardioverter-defibrillator recipients.. J Interv Card Electrophysiol..

[11] Turakhia M, Varosy P, Lee K (2007). Impact of renal function on survival in patients with implantable cardioverter-defibrillators.. Pace..

[12] Sakhuja R, Keebler M, Lai T, McLaughlin Gavin C, Thakur R, Bhatt D (2009). Meta-analysis of mortality in dialysis patients with an implantable cardioverter defibrillator.. Am J Cardiol..

[13] Genovesi S, Porcu L, Luise M (2015). Mortality, sudden death and indication for cardioverter defibrillator implantation in a dialysis population.. Int J Cardiol..

[14] Cuculich P, Sanchez J, Kerzner R (2007). Poor prognosis for patients with chronic kidney disease despite ICD therapy for the primary prevention of sudden death.. Pace..

[15] Pun P, Hellkamp A, Sanders G (2014). Primary prevention implantable cardioverter defibrillators in end-stage kidney disease patients on dialysis: a matched cohort study.. Nephrol Dial Transpl..

[16] Pun P, Al-Khatib S, Han J (2014). Implantable cardioverter-defibrillators for primary prevention of sudden cardiac death in CKD: a meta-analysis of patient-level data from 3 randomized trials.. Am J Kidney Dis..

[17] Waks J, Higgins A, Mittleman M, Buxton A (2015). Influence of renal function on mortality and ventricular arrhythmias in patients undergoing first implantable cardioverter-defibrillator generator replacement.. J Cardiovasc Electrophysiol..

[18] Eckart R, Gula L, Reynolds M, Shry E, Maisel W (2006). Mortality following defibrillator implantation in patients with renal insufficiency.. J Cardiovasc Electrophysiol..

[19] Robin J, Weinberg K, Tiongson J (2006). Renal dialysis as a risk factor for appropriate therapies and mortality in implantable cardioverter-defibrillator recipients.. Heart Rhythm..

[20] Fu L, Zhou Q, Zhu W (2017). Do implantable cardioverter defibrillators reduce mortality in patients with chronic kidney disease at all stages?. Int Heart J..

[21] Goldenberg I, Moss A, McNitt S (2006). Relations among renal function, risk of sudden cardiac death, and benefit of the implanted cardiac defibrillator in patients with ischemic left ventricular dysfunction.. Am J Cardiol..

[22] Herzog C, Li S, Weinhandl E, Strief J, Collins A, Gilbertson D (2005). Survival of dialysis patients after cardiac arrest and the impact of implantable cardioverter defibrillators.. Kidney Int..

[23] Mavrakanas T, Charytan D (2016). Cardiovascular complications in chronic dialysis patients.. Curr Opin Nephrol Hy..

[24] Charlson M, Pompei P, Ales K, MacKenzie C (1987). A new method of classifying prognostic comorbidity in longitudinal studies: development and validation.. J Chron Dis..

[25] Bardy G, Lee K, Mark D (2005). Amiodarone or an implantable cardioverter-defibrillator for congestive heart failure.. N Engl J Med..

[26] Tompkins C, Mclean R, Cheng A (2011). End-stage renal disease predicts complications in pacemaker and ICD implants.. J Cardiovasc Electrophysiol..

[27] Dasgupta A, Montalvo J, Medendorp S (2007). Increased complication rates of cardiac rhythm management devices in ESRD patients.. Am J Kidney Dis..

[28] Charytan D, Patrick A, Liu J (2011). Trends in the use and outcomes of implantable cardioverter-defibrillators in patients undergoing dialysis in the United States.. Am J Kidney Dis..

[29] Voigt A, Ezzeddine R, Barrington W (2004). Utilization of implantable cardioverter-defibrillators in survivors of cardiac arrest in the United States from 1996 to 2001.. J Am Coll Cardiol..

[30] Saba S, Ravipati L, Voigt A (2009). Recent trends in utilization of implantable cardioverter-defibrillators in survivors of cardiac arrest in the United States.. Pace..

[31] Ahn J, Waller JL, Baer SL (2019). Mortality risk after herpes zoster infection in end-stage renal disease patients.. Clin Kidney J..

